# Rapid diagnosis of mixed phenotype acute leukemia after identifying a blood histogram abnormality

**DOI:** 10.1016/j.plabm.2018.e00101

**Published:** 2018-05-12

**Authors:** Rie Saito, Hisayuki Yokoyama, Kuniaki Meguro, Yusuke Ohba, Yoshihiko Izumi, Shinichiro Takahashi

**Affiliations:** a Department of Clinical Laboratory, Tohoku Medical and Pharmaceutical University Hospital, Japan; b Department of Hematology, National Hospital Organization Sendai Medical Center, Japan; c Division of Laboratory Medicine, Faculty of Medicine, Tohoku Medical and Pharmaceutical University, 1-15-1, Fukumuro, Miyaginoku, Sendai 983-8536, Japan

**Keywords:** Mixed phenotype acute leukemia, Automatic blood cell analyzer, Histogram

## Abstract

A 38-year-old woman was suffering from back, right arm, and ankle joint pain, and visited our emergency department. Upon admission, the white blood cell (WBC) count was high (11,700/µL), and low numbers of red blood cells (2.21 × 10^6^/µL) and platelets (PLTs) (42,000/µL) were observed. A PLT histogram showed an abnormally shaped peak at around 20–30 fL, suggesting the presence of giant PLTs or PLT aggregation. The WBC histogram showed abnormal elevation at 35 fL and around 100 fL, suggesting abnormal cells including nucleated red blood cells. A peripheral blood smear was prepared, and morphology was examined. As a result, blasts (4%) including many orthochromatic erythroblasts (48/100 WBCs) were observed. Acute leukemia was suspected, and the patient was transferred the next day to a hospital with a hematology department. Bone marrow aspiration revealed that 99% of cells were blasts positive for B lymphoid lineage markers and myeloperoxidase. The patient was diagnosed with mixed phenotype lineage acute leukemia, treated immediately, and achieved remission. Thus, careful observation of histogram abnormalities of an automatic blood cell analyzer is important for rapid diagnosis of acute leukemia.

## 1 Introduction

Mixed phenotype lineage leukemia (MPAL) is a heterogenous category in the World Health Organization (WHO) classification that comprises acute leukemias with discrete admixed populations of myeloid and lymphoid blasts or extensive co-expression of lymphoid and myeloid markers in a single blast population [1]. Consensus criteria for MPAL were first published in the 4th edition of the WHO Classification of Tumours of Haematopoietic and Lymphoid Tissues and remained essentially unchanged in the 2016 update to the classification [2]. MPAL accounts for approximately 2% of acute leukemias in the WHO criteria. In most reports, the patients were initially treated with the local standard of care for B-acute lymphocytic leukemia (ALL), T-ALL, or acute myeloid leukemia (AML) based on which lineage appeared dominant by immunophenotyping and morphological evaluation. A recent review of stem cell transplantation for MPAL suggests that allogenic stem cell transplantation during first complete remission is beneficial [3]. Outcomes of MPAL patients are generally worse than those of AML and ALL patients [1]. Taken together, accurate diagnosis and prompt treatment are necessary for MPAL. An automated hematology analyzer provides blood cell histograms and, if it is interpreted well, has a good potential to provide diagnostically relevant information [4]. Here, we report a case of MPAL initially identified by a histogram abnormality of an automatic blood cell analyzer, which led to prompt treatment of the disease.

## 2 Case presentation

**Fig. 1:**
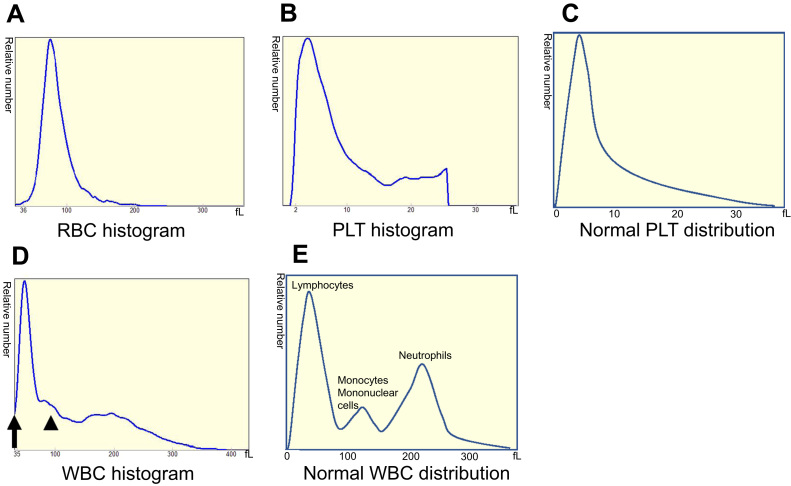
Histograms for (A) red blood cell (RBCs), (B) platelets (PLTs), and (D) white blood cells (WBCs). The arrow and arrowhead indicate abnormal elevation around 35 (matches up with the Y-axis) and 100 fL, respectively. (C) PLTs and (E) WBC histogram showing a normal PLTs and WBC distribution curve, respectively. The relative number is shown on the Y-axis and sizes of different blood cells are plotted on the X-axis.

**Fig. 2:**
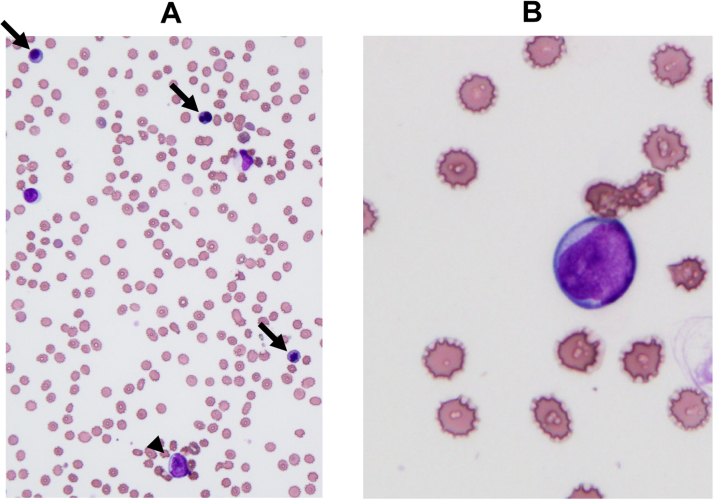
Morphological images of peripheral blood at initial diagnosis. Wright-Giemsa staining. (A) Morphological image with ×400 magnification. Arrows indicate erythroblasts and the arrowhead indicates a blast. (B) ×1000 magnification. The cell in the center is a blast.

**Fig. 3:**
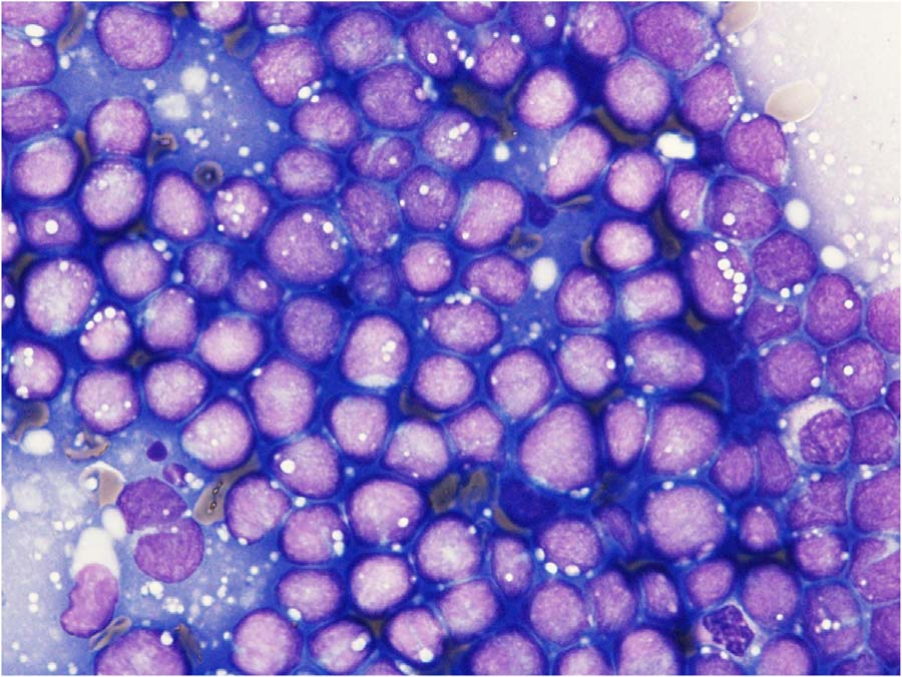
Morphological image of a bone marrow stamp at initial diagnosis with × 1000 magnification.

A 38-year-old woman suffering from back, right arm, and ankle joint pain for several months, had consulted several orthopedic, neurological and pain clinics. She was indicated to have anemia and advised to visit an internal medicine clinic. Before visiting another clinic, she visited the emergency department of our hospital at 11:30 P.M., Friday, because of severe back pain. Upon admission to our hospital, her white blood cell (WBC) count was slightly high (11,700/µL) and low numbers of red blood cells (RBCs) (2.21 × 10^6^/µL) and platelets (PLTs) (42,000/µL) were observed. Furthermore, high lactate dehydrogenase (2300 U/L), P-FDP (33.8 µg/mL), and d-dimer (14.22 µg/mL) were observed (Table 1). The histogram of an automated hematology analyzer (DxH 500; Beckman Coulter, CA) was abnormal. Although the RBC histogram was normal (Fig. 1A), the PLT histogram showed an abnormally shaped peak at around 20–30 fL (Fig. 1B), suggesting the presence of giant PLTs or PLT aggregation. The WBC histogram showed abnormal elevation at 35 fL (Fig. 1C, arrow) and around 100 fL (Fig. 1C, arrowhead), suggesting giant PLTs and abnormal cells including nucleated RBCs. A peripheral blood smear was prepared, and morphology was examined. As a result, blasts (4%) including many orthochromatic erythroblasts (48/100 WBCs) were observed (Fig. 2, Table 1). Acute leukemia was suspected and the patient was transferred the next day to Sendai Medical Center capable of acute leukemia treatment. Bone marrow was examined, and 99% of cells were blasts (Fig. 3) positive for CD10, CD19, CD24, CyCD22, CyCD79a, Cy µ, and myeloperoxidase as shown by flow cytometry (Table 2). The patient was diagnosed with MPAL, immediately treated by an acute lymphoid leukemia regimen (JALSG ALL202-O), and achieved remission. Genetic analysis revealed that there were no major (i.e. *major, minor-bcr-abl, PML-RARa, AML1-MTG8, DEK-CAN, NUP98-HOXA9, ETV6-AML1, E2A-PBX1, SIL/TAL1,* or *MLL gene* rearrangements or *FLT3-ITD*) abnormalities. However, G-banding analysis of bone marrow samples revealed that the patient had complex chromosomal aberrations (among 20 cells analyzed, three cells were A: 47, XX, +X, add(3)(q27), − 4, del(6)(q?), and +mar1, 13 cells were B: 47, idem, add(3)(q11.2), add(7)(q22), and del(7)(q?), and four cells were a type A-derived clone). Thereafter, the patient received an allogenic bone marrow transplantation. Until then, her remission was maintained and the patient is currently receiving immunosuppressive drugs as an outpatient.

**Table 1:** Laboratory findings at first visit.

Complete blood count						Biochemistry test			
WBC		11,700 /µL		RBC		T-Bil	0.8 mg/dL	CRP	12.53 mg/dL
Blasts			4%	Anisocytosis	(+)	AST	72 U/L	Na	134 mEq/L
Promyelocyte			1%	Deformation	(+)	ALT	28 U/L	K	4.3 mEq/L
Myelocyte			8%	Polychromatic	(+)	LD	2300 U/L	Cl	98 mEq/L
Metamyelocyte			6%	Nucleated RBC	48/100 WBC	ALP	267 U/L	Fib	531 mg/dL
Band			9%			γ-GTP	20 U/L	APTT	31.3 s
Seg			51%	PLT		ChE	228 U/L	PT	13.3 s
Eosinophil			2%	Giant platelets	(+)	CK	15 U/L	PT-INR	1.150
Basophil			0%			BUN	15 mg/dL	P-FDP	33.8 μg/mL
Monocyte			4%			Cr	0.39 mg/dL	D-dimer	14.22 μg/mL
Lymphocyte			15%			UA	6.3 mg/dL	TSH	2.380 μU/mL
RBC		2.21 × 10^6^ /µL				AMY	40 U/L	FreeT3	2.18 pg/mL
Hb		5.8 g/dL				lipase	31 U/L	FreeT4	1.35 ng/dL
Ht		18.7%				TP	6.8 g/dL		
MCV		84.8 fL							
MCH		26.2 pg							
MCHC		30.9 g/dL							
PLT		42,000 /µL

**Table 2:** Flow cytometric data.

Flow cytometry					
B-lymphoid		T-lymphoid		Myeloid	
CD10	99.1	CD2	0.9	CD13	6.4
CD19	85.2	CD3	0.5	CD14	1.8
CD24	98.1	CD4	0.5	CD15	0.5
Cy CD22	93.7	CD5	0.7	CD33	1.3
Cy CD79a	90.1	CD7	1.3	CD41	1.1
Cy μ	94.9	CD8	0.4	CD64	1.1
		Cy CD3	4.6	CD65	2.1
					
Other		TCR-αβ	0.6	MPO	95.2
CD34	18.3	TCR-γδ	0.5		
CD117	48.1				
TdT	89.3				
HLA-DR	99.3				

## 3 Discussion

During the night shift, it may not be possible to employ morphological examination of blood samples. However, our experience suggests that it is important to carefully observe the histograms of an automated blood cell analyzer, which led to prompt assessment of acute leukemia in this case. The complete blood count (CBC) histogram has suitable potential to provide diagnostically relevant information about several, like hemolytic anemia and idiopathic thrombocytopenic purpura disease processes, even before further advance examinations and investigations [4]. The ability of an automated blood cell analyzer to detect morphological abnormalities is extremely valuable. For example, identifying morphological abnormalities by a hematology analyzer is very efficient with high sensitivity (87%) and specificity (97%) by a SYSMEX NE-8000, which was reported in the mid 1990s [5]. In addition, it is well known that automated blood cell analyzers are good tools with the ability to detect large PLTs by histograms and flags [6]. Therefore, a good interpretation of the CBC count histogram provides a differential diagnosis at a very early stage before further studies.

## 4 Conclusion

Our experience highlighted that careful observation of a histogram abnormality of an automatic blood cell analyzer is important for rapid assessment of acute leukemia that requires prompt treatment.
